# Beyond Usual Pace: Quick Walking Speed and Brain Health Outcomes in the Framingham Heart Study

**DOI:** 10.64898/2026.10.13.26365066

**Published:** 2026-10-15

**Authors:** Anika Thomson, Sophia Lu, Vanessa M. Young, Haritha Vardhini Katragadda, Francis Kolo, Gabrielle Hromas, Monica Gireud-Goss, Mini E. Jacob, Joanne M. Murabito, Sudha Seshadri, Alexa Beiser

## Abstract

**INTRODUCTION:**

Stroke, dementia, and late-life depression share overlapping vascular pathophysiology, yet simple markers of shared neurological vulnerability across these conditions remain limited. Most gait research has relied on usual walking speed (UWS) alone, leaving it unclear whether quick walking speed (QWS) and gait adaptability (QWS minus UWS), relate to these conditions in the same way.

**METHODS:**

We examined associations of UWS, QWS, and gait adaptability with executive function, verbal memory, depressive symptoms, and incident stroke and dementia in the Framingham Heart Study Offspring cohort. A larger sample (n=2,308; mean age 61.5±9.2 years; 54% women) was used for cross-sectional and stroke analyses, and a dementia-free sample of 1,148 participants aged ≥60 years (mean age 68.5±5.8 years; 53% women) was used for analyses of incident dementia. We estimated associations per SD slower walking or smaller adaptability using linear, logistic, and Cox regression (10-year follow-up), overall and in women and men.

**RESULTS:**

Slower QWS and UWS were each associated with worse executive function, higher odds of high depressive symptoms (QWS OR 1.24, 95% CI 1.07-1.45; UWS OR 1.26, 95% CI 1.09-1.46), and greater hazard of incident stroke (QWS HR 1.30, 95% CI 1.03-1.64; UWS HR 1.36, 95% CI 1.11-1.65). Executive function and stroke associations were observed primarily in men (stroke QWS HR 1.55, 95% CI 1.11-2.17). For incident dementia, slower QWS reached significance (HR 1.34, 95% CI 1.10-1.63), while UWS did not (HR 1.20, 95% CI 0.98-1.46). Lower gait adaptability was associated with worse executive function overall and in women and men, and with incident dementia in women (HR 1.35, 95% CI 1.04-1.76), but not with depressive symptoms or stroke. No gait measure was associated with delayed recall.

**CONCLUSION:**

Slower QWS was associated with incident dementia; UWS was not. Adding a brief quick walking trial to routine gait assessment may identify risk that habitual-pace screening overlooks.

**Key Points:**

- Quick-pace walking, which adds one instruction to the usual-pace test, was associated with higher 10-year dementia risk, whereas the usual-pace estimate was smaller and not statistically significant.
- The margin between quick and usual pace, which reflects the capacity to accelerate on demand, was associated with executive function and, in women, with incident dementia, but not with stroke or depressive symptoms, unlike either pace on its own.

**Why does this paper matter?:**

- Gait speed is among the most widely collected measures in geriatric practice and aging research, yet it is almost always recorded at usual pace alone. This study shows that the margin between quick and usual pace carries a different pattern of brain health associations than either pace on its own, so adding one timed trial to an assessment clinicians already perform makes available information that habitual-pace testing cannot provide.

